# Nine eriophyoid mite species from Iran (Acari, Eriophyidae)

**DOI:** 10.3897/zookeys.143.2162

**Published:** 2011-11-01

**Authors:** Xiao-Feng Xue, Hussein Sadeghi, Xiao-Yue Hong, Samira Sinaie

**Affiliations:** 1 Department of Entomology, Nanjing Agricultural University, Nanjing, Jiangsu 210095, China; 2 Department of Plant Protection, Ferdowsi University of Mashhad, Razavi Khorasan, Iran

**Keywords:** New species, new records, Eriophyoidea, taxonomy, Iran

## Abstract

Nine eriophyoid mites, including two new species and five new records, from Iran are described and illustrated. They are *Aceria acroptiloni* Shevchenko & Kacalev, 1974, **rec. n.** on *Rhaponticum repens* (L.) Hidalgo (Asteraceae); *Aceria anthocoptes* (Nalepa, 1892), **rec. n.** on *Cirsium arvense* (L.) Scop. (Asteraceae); *Aceria lactucae* (Canestrini, 1893), **rec. n.** on *Lactuca virosa* L. (Asteraceae); *Aceria pulicaris* **sp. n.** on *Pulicaria gnaphalodes* (Vent.) Boiss. (Asteraceae); *Aceria tosichella* Keifer, 1969 on *Setaria viridis* (L.) Beauv. (Poaceae); *Eriophyes rotundae* Mohanasundaram, 1983 on *Cyperus rotundus* L. (Cyperaceae); *Aculops maroccensis* Keifer, 1972, **rec. n.** on *Mentha piperita* L. (Lamiaceae); *Aculus medicager* **sp. n.** on *Medicago sativa* L. (Leguminosae); *Tetra lycopersici* Xue & Hong, 2005, **rec. n.** on *Solanum nigrum* L. (Solanaceae).

## Introduction

During growth season 2010, field surveys were conducted by the second and forth authors in the north eastern provinces of Iran. A variety of locations mainly in Mashhad region were surveyed and sampled for potential eriophyoid symptoms and species presence. Among the eriophyoid mites that were identified, two species were found to be new to science and five species are reported for the first time from Iran. By this study, the total number of Eriophyoidea mites of Iran increased to 95 species (Xue et al. 2009; Xue et al. in press).

## Materials and methods

The specimens were recovered from plant materials by means of direct observations under a dissecting microscope. Collected mites were preserved in 70 % ethyl alcohol and later mounted or freshly collected specimens were placed in lactophenol solution for 5-7 days in room temperature then mounted in Hoyer’s medium. Slide mounted specimens were identified by the first and third authors. The morphological terminology used herein follows Lindquist (1996) and the generic classification is made according to Amrine et al. (2003). Slides were mounted and specimens were measured following de Lillo et al. (2010). Specimens were examined with a Zeiss A2 (Germany) research microscope with phase contrast and semi-schematic drawings were made. Photos of slide mounted mites were taken with the same microscope (100× oil immersion objective with 10× eyepieces), connected to a computer using Axiovision image analysis software. It was not possible to provide illustrations of the lateral views for some of the species described here because of the mounting position on slides. For each species, the holotype female measurement precedes the corresponding range for paratypes (given in parentheses). All measurements are in micrometers (μm), and are lengths when not otherwise specified.

## Taxonomy

### Family Eriophyidae Nalepa, 1898. Subfamily Eriophyinae Nalepa, 1898. Tribe Aceriini Amrine & Stansy, 1994. Genus Aceria Keifer, 1944

#### Aceria acroptiloni

Shevchenko & Kacalev, 1974 rec. n.

http://species-id.net/wiki/Aceria_acroptiloni

Figure 1

Aceria acroptiloni Shevchenko & Kacalev, 1974; Kacalev et al. 1974: 25–34, figures 1–4.
Aceria acroptiloni ; Amrine & Stasny 1994: 18.

##### Material examined.

2 females and 1 male (slide number IRAN210), from *Rhaponticum repens* (L.) Hidalgo (Asteraceae), Ferdowsi University campus, Mashhad, Razavi Khorasan Province, Iran, 36.3000°N, 59.5167°E, elevation 915m, 23-VIII-2010, coll. Samira Sinaie, deposited as slide mounted specimens in the Arthropod/Mite Collection of the Department of Entomology, NJAU, Jiangsu Province, China; 7females and 2 males (slide number 210), from *Rhaponticum repens* (L.) Hidalgo (Asteraceae), Shirvan, North Khorasan Province, Iran, 37.4500°N, 57.9000°E, elevation 1093m, 4-VI-2010, coll. Hussein Sadeghi, deposited as slides in the Department of Plant Protection, FUM, Iran.

**Figure:**
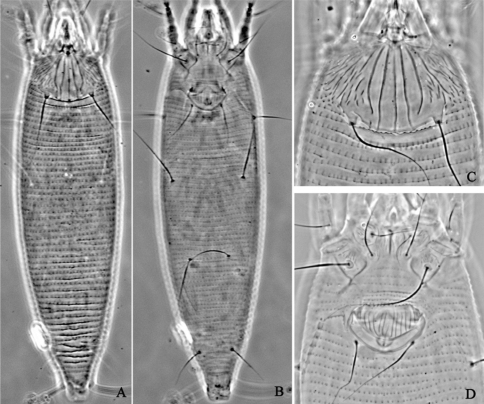
**Figure 1.** *Aceria acroptiloni* Shevchenko & Kacalev, 1974, rec. n. **A** dorsal view of female **B** ventral view of female **C** prodorsal shield **D** coxae and female genitalia.

##### Host.

*Rhaponticum repens* (L.) Hidalgo (Asteraceae).

##### Relation to host.

In flowers, flower buds, floral deformation.

##### Distribution.

Russia, Uzbekistan, Iran.

#### Aceria anthocoptes

(Nalepa, 1892) rec. n.

http://species-id.net/wiki/Aceria_anthocoptes

Figures 2 3

Phytoptus anthocoptes Nalepa 1892: 120.
Eriophyes anthocoptes ; Nalepa 1898: 42.
Aceria anthocoptes ; Roivainen 1950: 2.
Eriophyes (Aceria) anthocoptes ; Liro & Roivainen 1951: 81, figure 45–4.
Aceria anthocoptes ; Farkas 1965: 51, figure 35a–c.
Aceria anthocoptes ; Amrine & Stasny 1994: 22.
Aceria anthocoptes ; Skoracka et al. 2005: 42.

##### Description.

Female (n = 11, dorsal view): Body vermiform, 256 (256–282), 67 (67–68) wide; light yellow. **Gnathosoma** 22 (22–23), projecting obliquely downwards, pedipalp coxal seta (*ep*) 3 (2–3), dorsal pedipalp genual seta (*d*) 5 (5–6), cheliceral stylets 20 (20–21). **Prodorsal shield** 28 (28–33), 37 (37–38) wide, median, admedian and submedian lines complete and parallel, with many short lines and granules at lateral; anterior shield lobe absent. Scapular tubercles near rear shield margin, 28 (28–29) apart, scapular setae (*sc*) 52 (52–56), projecting posteriorly. **Coxigenital region** with 7 (7–9) microtuberculated annuli. Coxisternal plates with short lines, anterolateral setae on coxisternum **І** (*1b*) 7 (7–8), 12 (12–13) apart, proximal setae on coxisternum **І** (*1a*) 25 (25–27), 8 (8–10) apart, proximal setae on coxisternum **ІІ** (*2a*) 58 (53–58), 25 (25–26) apart, tubercles *1b* and *1a* apart 7 (6–7), tubercles *1a* and *2a* 8 (8–8) apart. Prosternal apodeme 7 (7–8). **Legs** with usual series of setae. Leg **І** 38 (38–41), femur 12 (12–13), basiventral femoral seta (*bv*) 12 (12–13); genu 5 (5–6), antaxial genual seta (*l’’*) 30 (30–33); tibia 6 (6–7), paraxial tibial seta (*l’*) 5 (5–6), located at 1/3 from dorsal base; tarsus 8 (7–8), seta *ft’* 18 (18–19), seta *ft’’* 26 (26–27), seta *u’* 5 (5–6); tarsal empodium (*em*) 7 (7–8), simple, 5-rayed, tarsal solenidion (*ω*) 8 (8–9), knobbed. Leg **ІІ** 37 (37–39), femur 9 (9–10), basiventral femoral seta (*bv*) 13 (13–14); genu 4 (4–5), antaxial genual seta (*l’’*) 10 (10–12); tibia 6 (5–6); tarsus 7 (6–7), seta *ft’* 8 (8–9), seta *ft’’* 28 (28–30), seta *u’* 5 (5–6); tarsal empodium (*em*) 7 (7–8), simple, 5-rayed, tarsal solenidion (*ω*) 11 (11–12), knobbed. **Opisthosoma:** opisthosoma dorsally with 71 (71–75) annuli, with elliptical microtubercles on rear annular margins, ventrally with 79 (79–83) annuli, with round microtubercles on rear annular margins. Setae *c2* 23 (23–25) on ventral annulus 15 (15–16), 62 (57–62) apart; setae *d* 73 (73–76) on ventral annulus 29 (28–29), 50 (45–50) apart; setae *e* 23 (23–25) on ventral annulus 46 (45–46), 30 (22–30) apart; setae *f* 32 (32–35) on 7th ventral annulus from rear, 23 (23–23) apart. Setae *h1* 5 (5–6), *h2* 96 (96–98). **Female genitalia** 15 (15–16), 25 (25–26) wide, coverflap with 14 longitudinal ridges, setae *3a* 23 (23–25), 21 (18–21) apart.

**Male**. Not seen.

**Figure:**
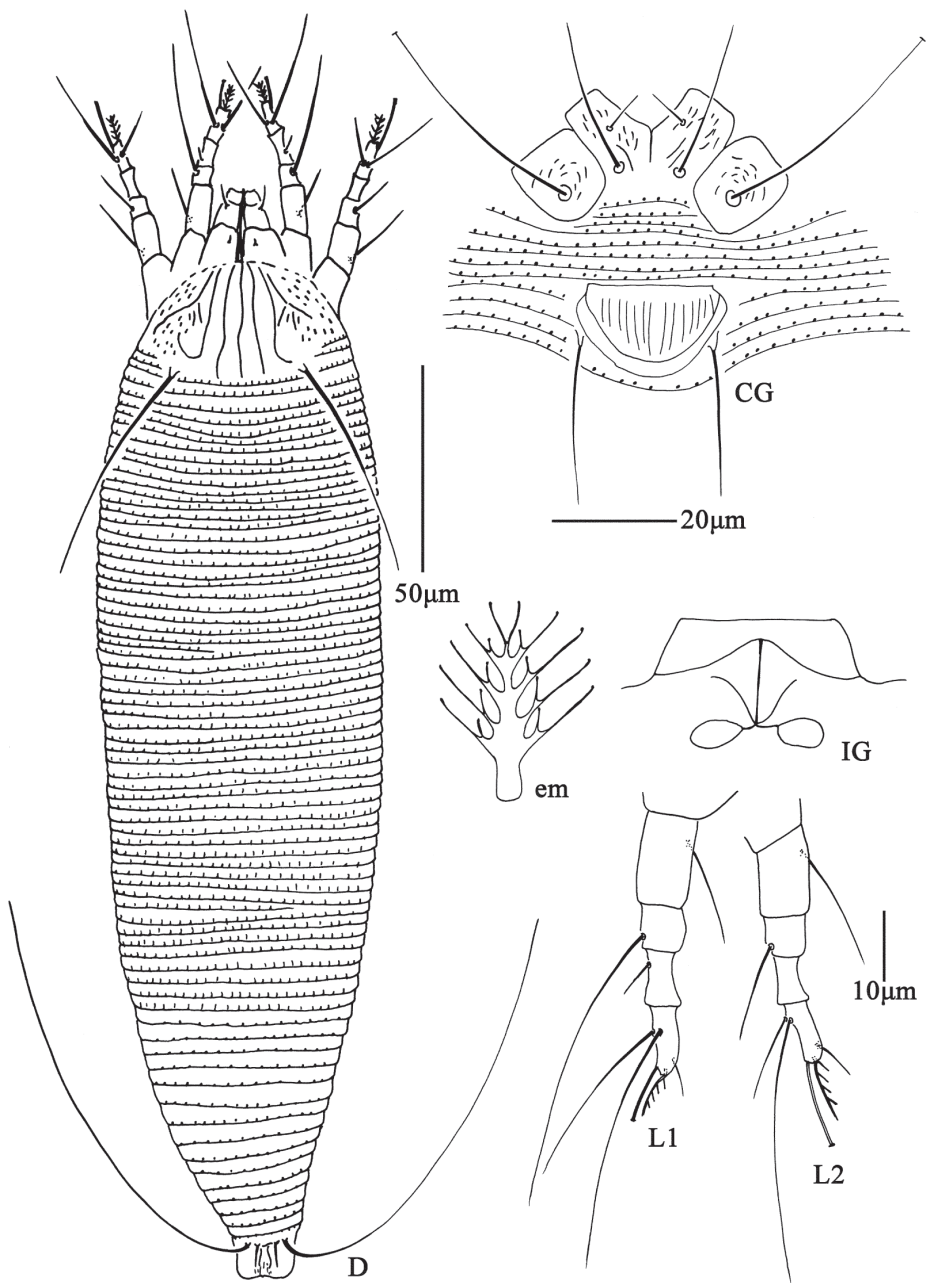
**Figure 2.** *Aceria anthocoptes* (Nalepa, 1892), rec. n. **D** dorsal view of female **CG** coxae and female genitalia **em** empodium **L1** leg **І** **L2** leg **ІІ** **IG** female internal genitalia.

**Figure:**
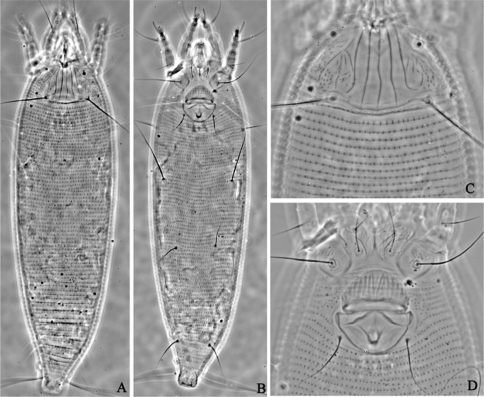
**Figure 3.** *Aceria anthocoptes* (Nalepa, 1892), rec. n. **A** dorsal view of female **B** ventral view of female **C** prodorsal shield **D** coxae and female genitalia.

##### Material examined.

3females (slide number IRAN212), from *Cirsium arvense* (L.) Scop. (Asteraceae), Torghabeh, Mashhad, Razavi Khorasan Province, Iran, 36.3167°N, 59.3500°E, 26-VIII-2010, elevation 980m & Ferdowsi University campus, Mashhad, Razavi Khorasan Province, Iran, 36.3000°N, 59.5167°E, elevation 915m, 6-IX-2010, coll. Samira Sinaie, deposited as slide mounted specimens in the Arthropod/Mite Collection of the Department of Entomology, NJAU, Jiangsu Province, China; 8females (slide number 212), from *Cirsium arvense* (L.) Scop. (Asteraceae), Torghabeh, Mashhad, Razavi Khorasan Province, Iran, 36.3167°N, 59.3500°E, 26-VIII-2010, elevation 980m, coll. Samira Sinaie, deposited as slides in the Department of Plant Protection, FUM, Iran.

##### Host.

*Cirsium arvense* (L.) Scop., *Cirsium calcareum* (M.E. Jones) Woot. & Standl., *Cirsium canescens* Nutt., *Cirsium helenioides* (L.) Hill, *Cirsium scariosum* Nutt., *Cirsium scopulorum* (Greene) Cockerell ex Daniels, *Cirsium undulatum* (Nutt.) Spreng., *Cirsium vulgare* (Savi) Tenore (Asteraceae); *Lolium perenne* L. (Poaceae).

##### Relation to host.

Leaf curl, erineum, rust, vagrant.

##### Distribution.

Iran, Austria, Bulgaria, Croatia, Denmark, France, Finland, Germany, Hungary, Italy, Poland, Serbia, Sweden, Turkey, USA.

#### Aceria lactucae

(Canestrini, 1893) rec. n.

http://species-id.net/wiki/Aceria_lactucae

Figures 4 5

Phytoptus lactucae Canestrini 1893: 153.
Eriophyes lactucae ; Nalepa 1898: 43.
Aceria lactucae ; Farkas 1965: 42, figure 34c.
Aculus lactucae ; Amrine & Stasny 1994: 135.
Vasates lactucae ; Petanovic & Stankovic 1999: 80.

##### Description.

Female (n = 14, dorsal view): Body vermiform, 273 (242–273), 62 (62–63) wide; light yellow. **Gnathosoma** 20 (20–21), projecting obliquely downwards, pedipalp coxal seta (*ep*) 2 (2–3), dorsal pedipalp genual seta (*d*) 6 (6–7), cheliceral stylets 18 (17–18). **Prodorsal shield** 35 (35–37), 45 (45–46) wide, median, admedian and submedian lines complete and parallel, with many short lines at lateral; anterior shield lobe acuminate. Scapular tubercles near rear shield margin, 25 (25–26) apart, scapular setae (*sc*) 42 (45–45), projecting posteriorly. **Coxigenital region** with 8 (8–9) microtuberclated annuli. Coxisternal plates with short lines, anterolateral setae on coxisternum **І** (*1b*) 14 (14–16), 13 (12–13) apart, proximal setae on coxisternum **І** (*1a*) 28 (28–30), 11 (10–11) apart, proximal setae on coxisternum **ІІ** (*2a*) 42 (42–45), 26 (26–27) apart, tubercles *1b* and *1a* apart 7 (6–7), tubercles *1a* and *2a* 8 (8–9) apart. Prosternal apodeme 10 (10–11). **Legs** with usual series of setae. Leg **І** 40 (40–42), femur 10 (9–10), basiventral femoral seta (*bv*) 13 (13–14); genu 5 (4–5), antaxial genual seta (*l’’*) 31 (31–33); tibia 8 (7–8), paraxial tibial seta (*l’*) 10 (10–11), located at 1/3 from dorsal base; tarsus 7 (7–8), seta *ft’* 19 (16–19), seta *ft’’* 27 (27–28), seta *u’* 7 (7–8); tarsal empodium (*em*) 9 (9–10), simple, 5-rayed, tarsal solenidion (*ω*) 10 (10–11), tapered. Leg **ІІ** 36 (36–38), femur 8 (7–8), basiventral femoral seta (*bv*) 13 (13–14); genu 4 (4–5), antaxial genual seta (*l’’*) 15 (14–15); tibia 6 (5–6); tarsus 7 (6–7), seta *ft’* 12 (12–13), seta *ft’’* 31 (31–33), seta *u’* 6 (5–6); tarsal empodium (*em*) 10 (10–11), simple, 5-rayed, tarsal solenidion (*ω*) 11 (11–12), tapered. **Opisthosoma:** opisthosoma dorsally with 68 (68–72) annuli, with round obscure microtubercles on rear annular margins, ventrally with 78 (78–80) annuli, with round microtubercles on rear annular margins. Setae *c2* 30 (29–30) on ventral annulus 15 (15–16), 61 (58–61) apart; setae *d* 62 (62–66) on ventral annulus 27 (27–29), 46 (46–48) apart; setae *e* 21 (21–25) on ventral annulus 44 (44–45), 26 (26–27) apart; setae *f* 27 (27–30) on 7th ventral annulus from rear, 20 (20–21) apart. Setae *h1* 5 (5–6), *h2* 83 (83–85). **Female genitalia** 20 (20–22), 25 (25–26) wide, coverflap with 12 longitudinal ridges, setae *3a* 23 (23–25), 17 (17–18) apart.

**Male**: Not seen.

**Figure:**
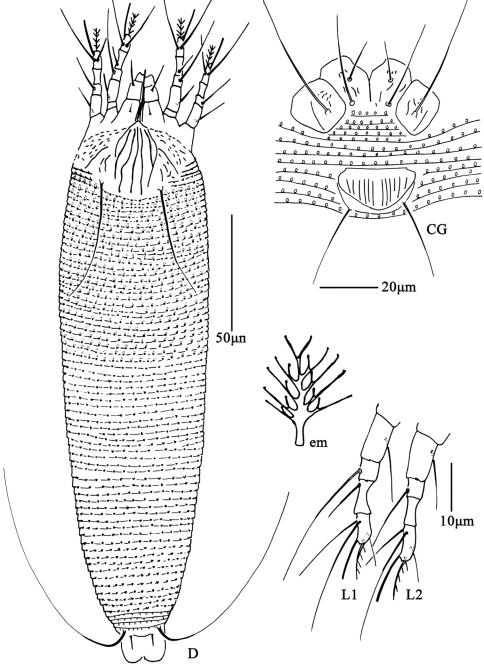
**Figure 4.** *Aceria lactucae* (Canestrini, 1893), rec. n. **D** dorsal view of female **CG** coxae and female genitalia **em** empodium **L1** leg **І** **L2** leg **ІІ**.

**Figure:**
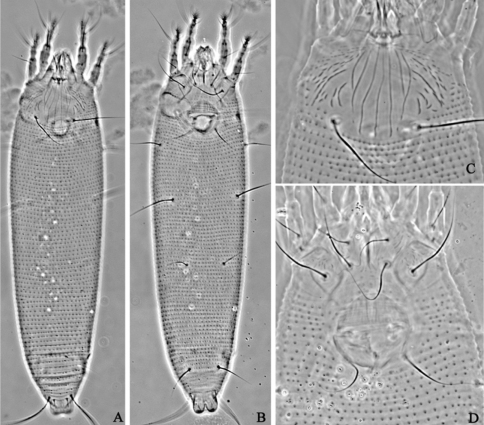
**Figure 5.** *Aceria lactucae* (Canestrini, 1893), rec. n. **A** dorsal view of female **B** ventral view of female **C** prodorsal shield **D** coxae and female genitalia.

##### Material examined.

3females (slide number IRAN216), from *Lactuca virosa* L. (Asteraceae), Kang, Mashhad, Razavi Khorasan Province, Iran, 36.3167°N, 59.2333°E, elevation 1050m, 26-VII-2010 & Ferdowsi University campus, Mashhad, Razavi Khorasan Province, Iran, 36.3000°N, 59.5167°E, 20-X-2010, coll. Samira Sinaie, deposited as slide mounted specimens in the Arthropod/Mite Collection of the Department of Entomology, NJAU, Jiangsu Province, China; 11females (slide number 216), from *Lactuca virosa* L. (Asteraceae), Ferdowsi University campus, Mashhad, Razavi Khorasan Province, Iran, 36.3000°N, 59.5167°E, elevation 915m, 9-X-2010, coll. Samira Sinaie, deposited in the Department of Plant Protection, FUM, Iran.

##### Host.

*Lactuca saligna* L., *Lactuca serriola* L., *Lactuca virosa* L. (Asteraceae).

##### Relation to host.

Bract and leaf deformation.

##### Distribution.

Iran; Hungary; Italy.

#### Aceria pulicaris sp. n.

urn:lsid:zoobank.org:act:1C9F694D-397D-4919-A594-462CAB23BFE7

http://species-id.net/wiki/Aceria_pulicaris

**Figure F7:**
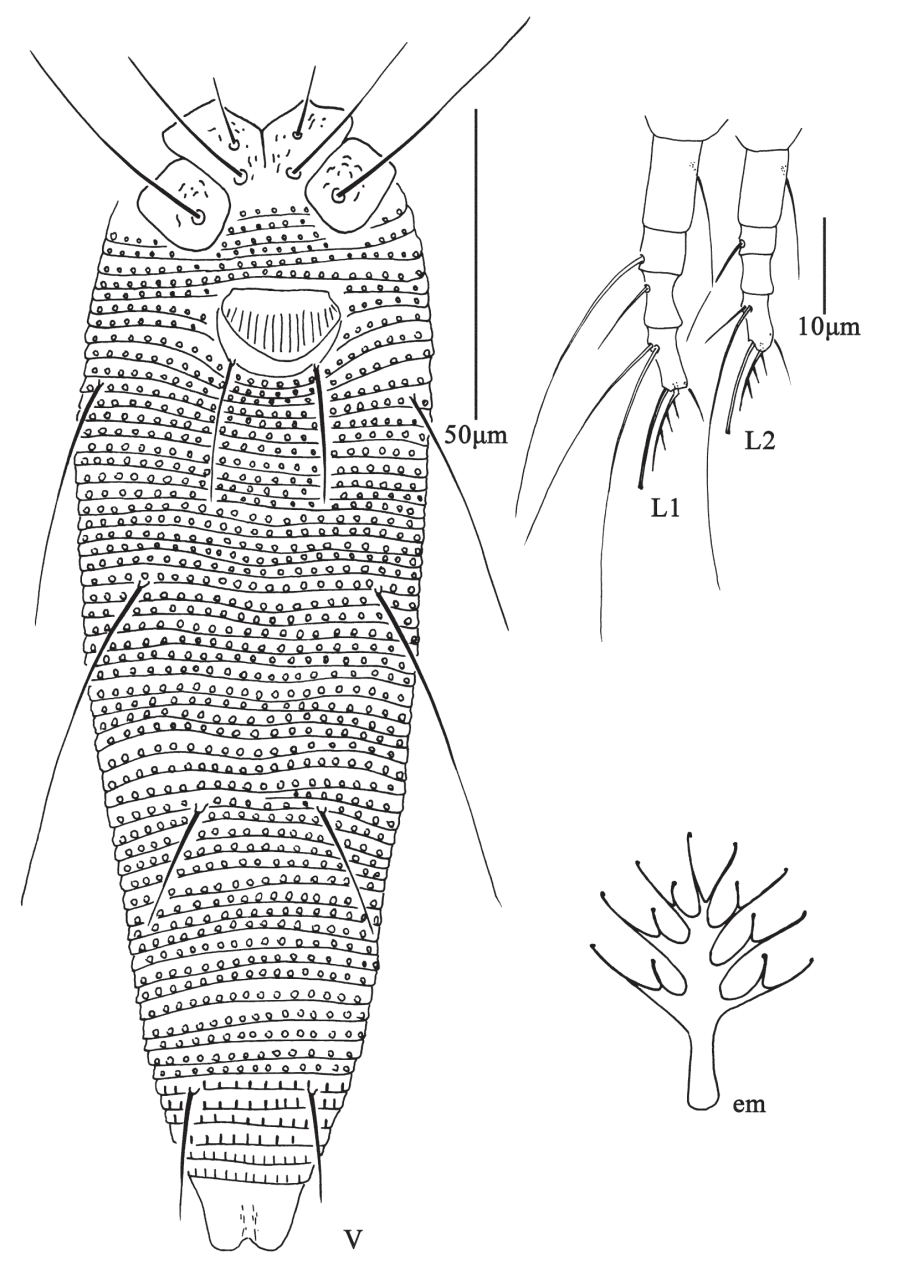
**Figure 7.** *Aceria pulicaris* sp. n. **V** ventral view of female **em** empodium **L1** leg **І** **L2** leg **ІІ**.

Figures 6 [Fig F7] 8

##### Description.

Female (n = 10, dorsal view): Body vermiform, 203 (203–223), 58 (55–58) wide; light yellow. **Gnathosoma** 25 (25–26), projecting obliquely downwards, pedipalp coxal seta (*ep*) 3 (2–3), dorsal pedipalp genual seta (*d*) 5 (5–7), cheliceral stylets 18 (18–23). **Prodorsal shield** 31 (31–35), 35 (35–36) wide, median, admedian and submedian lines complete and parallel, between median and admedian lines with some short lines, prodorsal shield with many short lines at lateral; anterior shield lobe acuminate. Scapular tubercles near rear shield margin, 22 (22–24) apart, scapular setae (*sc*) 43 (43–44), projecting posteriorly. **Coxigenital region** with 6 (6–7) microtuberclated annuli. Coxisternal plates with short lines and granules, anterolateral setae on coxisternum **І** (*1b*) 11 (8–11), 10 (10–11) apart, proximal setae on coxisternum **І** (*1a*) 29 (27–29), 7 (7–8) apart, proximal setae on coxisternum **ІІ** (*2a*) 45 (45–48), 21 (21–22) apart, tubercles *1b* and *1a* apart 5 (5–6), tubercles *1a* and *2a* 7 (7–8) apart. Prosternal apodeme 6 (6–7). **Legs** with usual series of setae. Leg **І** 38 (38–41), femur 10 (10–11), basiventral femoral seta (*bv*) 14 (14–15); genu 5 (5–6), antaxial genual seta (*l’’*) 32 (32–33); tibia 5 (5–6), paraxial tibial seta (*l’*) 8 (7–8), located at 1/3 from dorsal base; tarsus 8 (7–8), seta *ft’* 24 (24–25), seta *ft’’*30 (28–30), seta *u’* 5 (5–6); tarsal empodium (*em*) 6 (6–7), simple, 4-rayed, tarsal solenidion (*ω*) 10 (10–11), slightly knobbed. Leg **ІІ** 33 (33–35), femur 8 (8–9), basiventral femoral seta (*bv*) 12 (12–13); genu 3 (3–4), antaxial genual seta (*l’’*) 12 (12–13); tibia 4 (4–5); tarsus 7 (7–8), seta *ft’* 6 (6–7), seta *ft’’* 30 (30–31), seta *u’* 5 (5–6); tarsal empodium (*em*) 6 (6–7), simple, 4-rayed, tarsal solenidion (*ω*) 10 (10–11), slightly knobbed. **Opisthosoma:** opisthosoma dorsally with 66 (66–69) annuli, with round microtubercles on rear annular margins, ventrally with 64 (64–68) annuli, with round microtubercles on rear annular margins. Setae *c2* 40 (40–43) on ventral annulus 10 (10–11), 55 (55–56) apart; setae *d* 58 (58–62) on ventral annulus 24 (24–25), 38 (38–40) apart; setae *e* 21 (21–23) on ventral annulus 40 (40–41), 20 (20–23) apart; setae *f* 22 (22–23) on 6th ventral annulus from rear, 18 (18–20) apart. Setae *h1* 3 (3–4), *h2* 63 (63–66). **Female genitalia** 15 (15–16), 20 (20–21) wide, coverflap with 16 longitudinal ridges, setae *3a* 21 (21–22), 13 (13–15) apart.

**Male**: Unknown.

**Figure:**
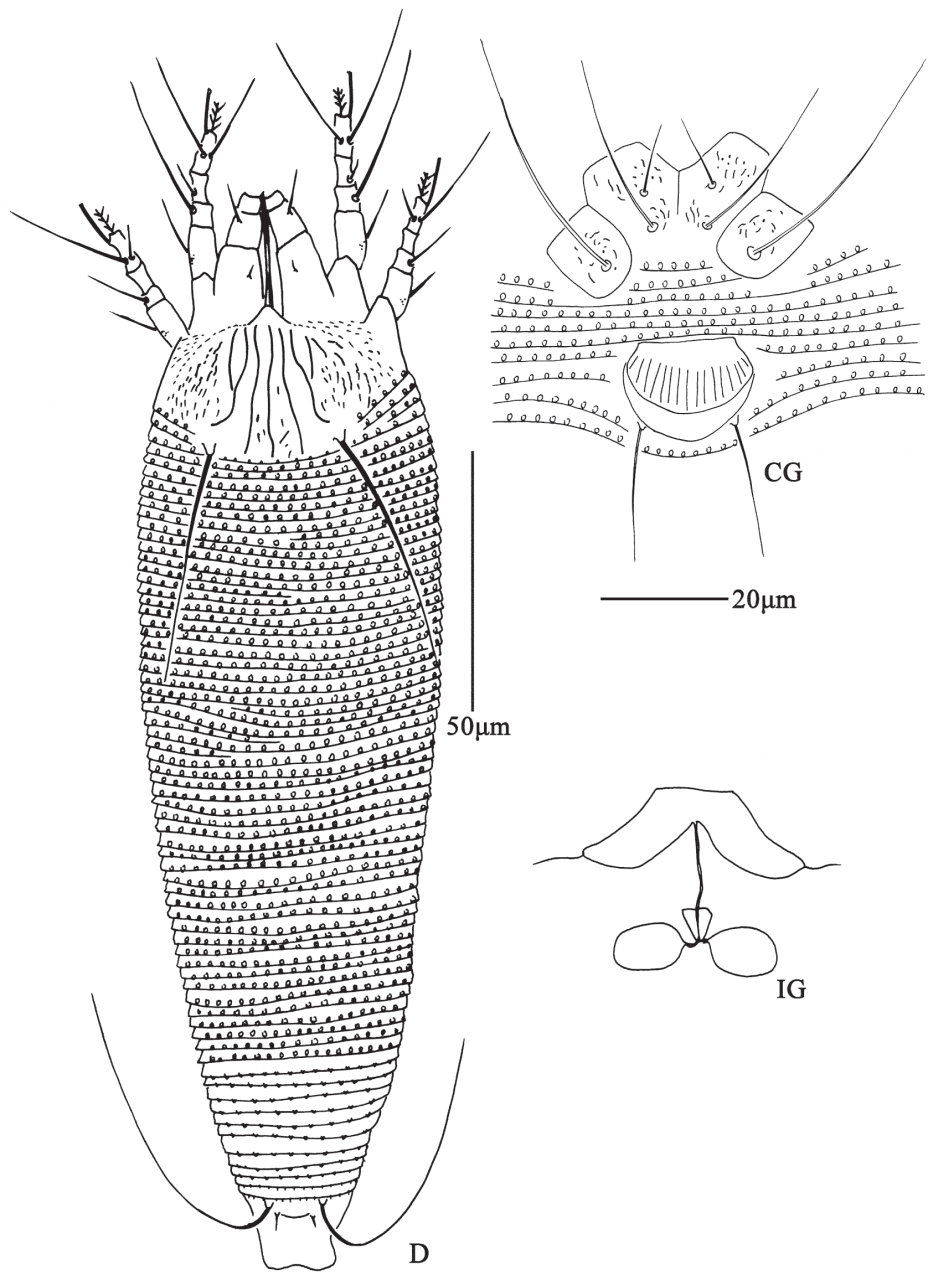
**Figure 6.** *Aceria pulicaris* sp. n. **D** dorsal view of female **CG** coxae and female genitalia **IG** female internal genitalia.

**Figure:**
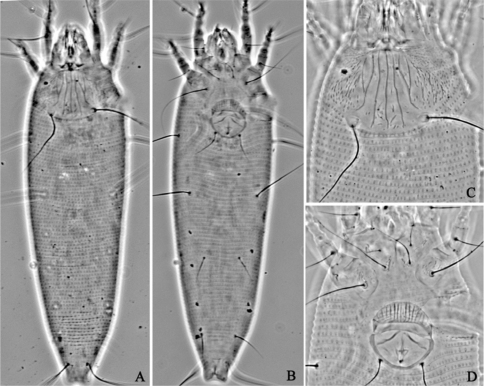
**Figure 8.** *Aceria pulicaris* sp. n. **A** dorsal view of female **B** ventral view of female **C** prodorsal shield **D** coxae and female genitalia.

##### Type material.

Holotype, female (slide number IRAN207, marked Holotype), from *Pulicaria gnaphalodes* (Vent.) Boiss. (Asteraceae), Ferdowsi University campus, Mashhad, Razavi Khorasan Province, Iran, 36.3000°N, 59.5167°E, elevation 915m, 21-VIII-2010, coll. Samira Sinaie, deposited as slide mounted specimens in the Arthropod/Mite Collection of the Department of Entomology, NJAU, Jiangsu Province, China. Paratypes, 2 females (slide number IRAN207), with the same data as holotype; 7females (slide number 207), from *Pulicaria gnaphalodes* (Vent.) Boiss. (Asteraceae), Ferdowsi University campus, Mashhad, Razavi Khorasan Province, Iran, 36.3000°N, 59.5167°E, elevation 915m, 21-VIII-2010, coll. Samira Sinaie, deposited as slides in the Department of Plant Protection, FUM, Iran.

##### Relation to host.

Vagrant.

##### Etymology.

The specific designation *pulicaris* is from the generic name of host plant, *Pulicaria*.

##### Differential diagnosis.

This species is similar to *Aceria lactucae* (Canestrini, 1893), but can be differentiated from the latter by female genital coverflap with 16 ridges (female genital coverflap with 12 ridges in *Aceria lactucae*), empodium 4-rayed (empodium 5-rayed in *Aceria lactucae*), between median and admedian lines with short lines on prodorsal shield (between median and admedian lines smooth in *Aceria lactucae*).

#### Aceria tosichella

Keifer, 1969

http://species-id.net/wiki/Aceria_tosichella

Figure 9

Aceria tosichella Keifer, 1969: 1–2, pl.1.
Aceria tritici Shevtchenko, 1970; Shevtchenko et al. 1970: 224–235, figures 2–4.
Aceria tosichella ; Amrine & Stasny 1994: 92.
Aceria tosichella ; Hong & Zhang 1996: 28.
Aceria tosichella ; Baker et al. 1996: 318, figure 573.
Aceria tosichella ; Skoracka 2005: 64–66.
Aceria tosichella ; Hong et al. 2006: 231.
Aceria tosichella ; Song et al. 2008: 14.
Aceria tosichella ; Ripka 2008: 154.
Aceria tosichella ; Xue et al. 2009: 466.
Aceria tosichella ; Pereira et al. 2009: 539–542.

##### Material examined.

3females (slide number IRAN209), from *Setaria viridis* (L.) Beauv. (Poaceae), Ferdowsi University campus, Mashhad, Razavi Khorasan Province, Iran, 36.3000°N, 59.5167°E, elevation 915m, 21-VIII-2010, coll. Hussein Sadeghi, deposited as slide mounted specimens in the Arthropod/Mite Collection of the Department of Entomology, NJAU, Jiangsu Province, China; 11females (slide number 209), from *Setaria viridis* (L.) Beauv. (Poaceae), Ferdowsi University campus, Mashhad, Razavi Khorasan Province, Iran, 36.3000°N, 59.5167°E, elevation 915m, 21-VIII-2010, coll. Samira Sinaie, deposited in the Department of Plant Protection, FUM, Iran.

##### Host.

*Avena sativa* L., *Hordeum vulgare* L., *Pennisetum* sp. Rich, *Secale cereale* L., *Setaria viridis* (L.) Beauv., *Sorghum* sp. Moench, *Triticum aestivum* L., *Zea mays* L. (Poaceae).

**Figure:**
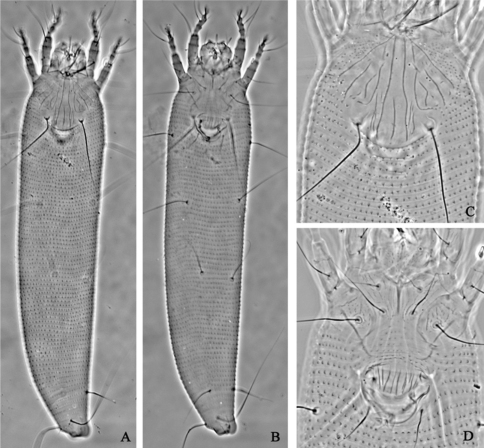
**Figure 9.** *Aceria tosichella* Keifer, 1969 **A** dorsal view of female **B** ventral view of female **C** prodorsal shield **D** coxae and female genitalia.

##### Relation to host.

Vagrant, often causing leaf curl, virus transmission.

##### Distribution.

Asia; Australia; Brazil; Canada; Europe; Iran; Italy; Mexico; Poland; Russia; Serbia; USA.

**Tribe Eriophyini Nalepa, 1898**

**Genus *Eriophyes* von Siebold, 185*1***

#### Eriophyes rotundae

Mohanasundaram, 1983

http://species-id.net/wiki/Eriophyes_rotundae

Figure 10

Eriophyes rotundae Mohanasundaram 1983: 263–265, figure 1.
Eriophyes rotundae ; Amrine & Stasny 1994: 208.

##### Material examined.

3females (slide number IRAN218), from *Cyperus rotundus* L. (Cyperaceae), Torogh, Mashhad, Razavi Khorasan Province, Iran, 36.2333°N, 59.6000°E, 22-X-2010, elevation 920m, coll. Samira Sinaie, deposited as slide mounted specimens in the Arthropod/Mite Collection of the Department of Entomology, NJAU, Jiangsu Province, China; 16females (slide number 218), from *Cyperus rotundus* L. (Cyperaceae), Torogh, Mashhad, Razavi Khorasan Province, Iran, 36.2333°N, 59.6000°E, 22-X-2010, elevation 920m, coll. Samira Sinaie, deposited in the Department of Plant Protection, FUM, Iran.

**Figure:**
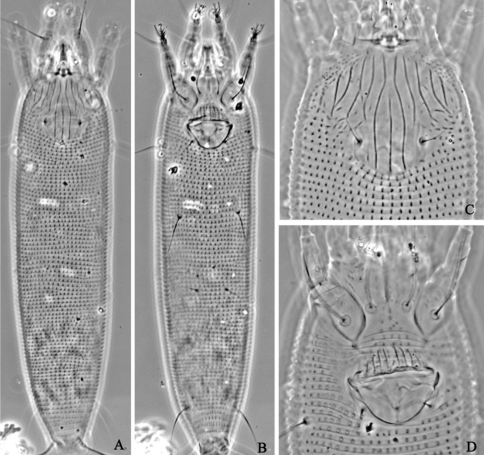
**Figure 10.** *Eriophyes rotundae* Mohanasundaram, 1983 **A** dorsal view of female **B** ventral view of female **C** prodorsal shield **D** coxae and female genitalia.

##### Host.

*Cyperus rotundus* L. (Cyperaceae).

##### Relation to host.

Vagrant.

##### Distribution.

India, Iran.

**Subfamily Phyllocoptinae Nalepa, 1892**

**Tribe Anthocoptini Amrine & Stasny, 1994**

**Genus *Aculops* Keifer, 196*6***

#### Aculops maroccensis

Keifer, 1972 rec. n.

http://species-id.net/wiki/Aculops_maroccensis

Figure 11

Aculops maroccensis Keifer 1972: 3, pl. 2.
Aculops maroccensis ; Amrine & Stasny 1994: 108.

##### Material examined.

3females (slide number IRAN205), from *Mentha piperita* L. (Lamiaceae), Golmakan, Mashhad, Razavi Khorasan Province, Iran, 36.4833°N, 59.1500°E, elevation 945m, 13-VIII-2010, coll. Samira Sinaie, deposited as slide mounted specimens in the Arthropod/Mite Collection of the Department of Entomology, NJAU, Jiangsu Province, China; 15 females (slide number 205), from *Mentha piperita* L. (Lamiaceae), Golmakan, Mashhad, Razavi Khorasan Province, Iran, 36.4833°N, 59.1500°E, elevation 945m, 13-VIII-2010, coll. Samira Sinaie, deposited in the Department of Plant Protection, FUM, Iran.

**Figure:**
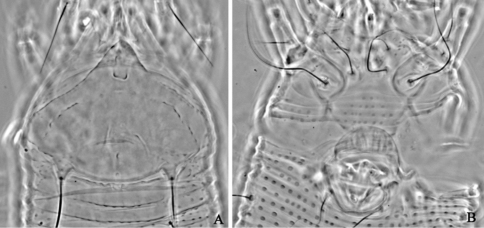
**Figure 11.** *Aculops maroccensis* Keifer, 1972, rec. n. **A** prodorsal shield **B** coxae and female genitalia.

##### Host.

*Mentha piperita* L. (Lamiaceae).

##### Relation to host.

Vagrant.

##### Distribution.

Morocco, Iran.

### Genus Aculus Keifer, 1959

#### Aculus medicager sp. n.

urn:lsid:zoobank.org:act:D46F68D0-77E6-4419-94EB-3BB509384865

http://species-id.net/wiki/Aculus_medicager

**Figure F13:**
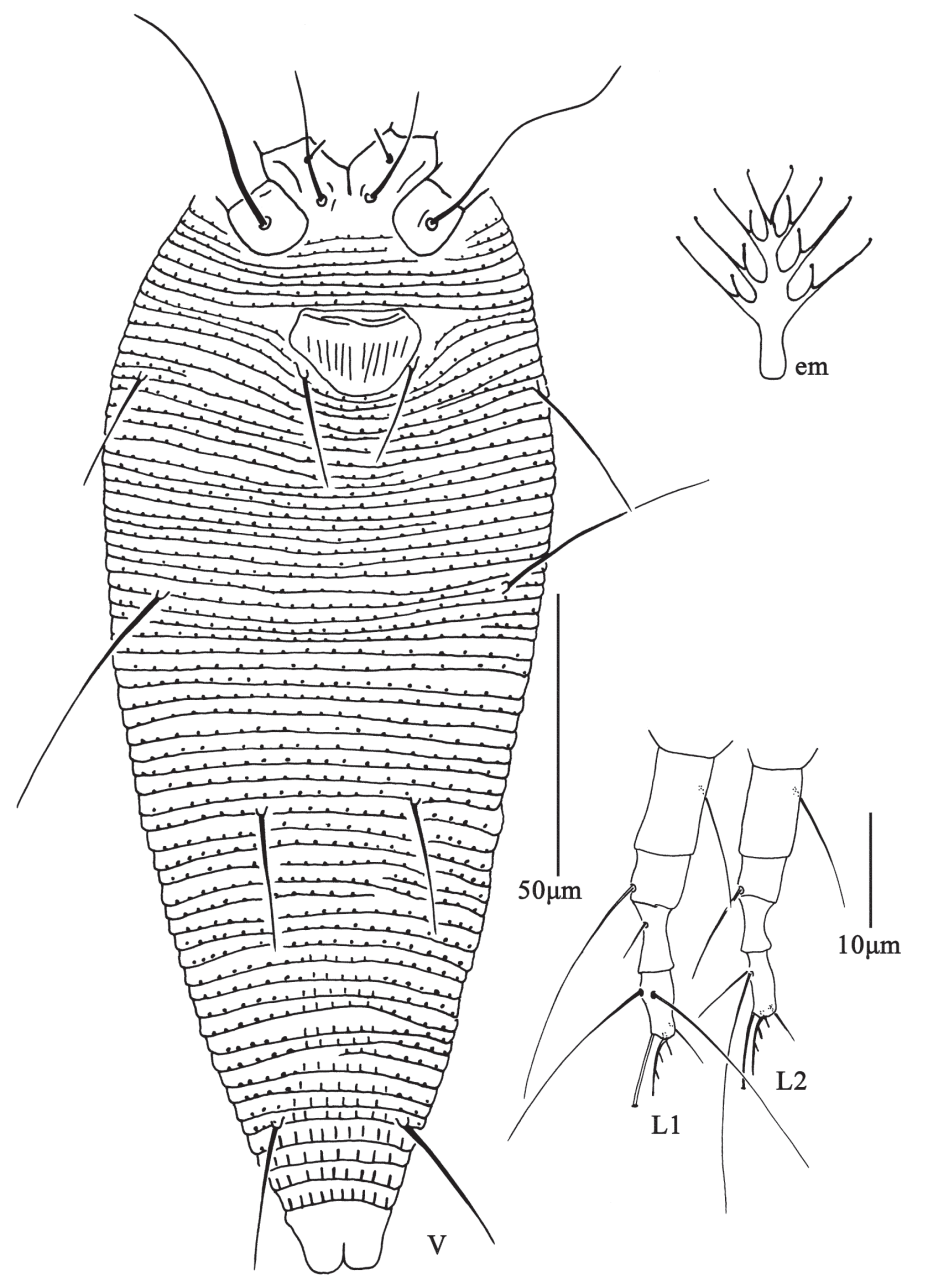
**Figure 13.** *Aculus medicager* sp. n. **V** ventral view of female **em** empodium **L1** leg **І** **L2** leg **ІІ**.

Figures 12 [Fig F13] 14

##### Description.

Female (n = 14, dorsal view): Body fusiform, 218 (218–233), 72 (69–72) wide; light yellow. **Gnathosoma** 24 (24–25), projecting obliquely downwards, pedipalp coxal seta (*ep*) 3 (2–3), dorsal pedipalp genual seta (*d*) 7 (6–7), cheliceral stylets 22 (22–23). **Prodorsal shield** 42 (42–43), 52 (50–52) wide, median and admedian lines absent, submedian lines incomplete, prodorsal shield with many granules at lateral; anterior shield lobe broad. Scapular tubercles near rear shield margin, 37 (37–40) apart, scapular setae (*sc*) 17 (17–18), projecting posteriorly. **Coxigenital region** with 7 (6–7) microtuberclated annuli. Coxisternal plates with few short lines, anterolateral setae on coxisternum **І** (*1b*) 4 (4–7), 14 (13–14) apart, proximal setae on coxisternum **І** (*1a*) 23 (23–28), 7 (7–8) apart, proximal setae on coxisternum **ІІ** (*2a*) 47 (45–47), 27 (25–27) apart, tubercles *1b* and *1a* apart 6 (5–6), tubercles *1a* and *2a* 11 (10–11) apart. Prosternal apodeme 6 (6–7). **Legs** with usual series of setae. Leg **І** 37 (37–40), femur 9 (9–10), basiventral femoral seta (*bv*) 14 (14–15); genu 5 (5–6), antaxial genual seta (*l’’*) 24 (24–25); tibia 8 (7–8), paraxial tibial seta (*l’*) 4 (4–5), located at 1/3 from dorsal base; tarsus 7 (7–8), seta *ft’* 22 (22–23), seta *ft’’* 27 (27–30), seta *u’* 5 (5–6); tarsal empodium (*em*) 7 (6–7), simple, 4-rayed, tarsal solenidion (*ω*) 8 (8–9), slightly knobbed. Leg **ІІ** 34 (34–37), femur 9 (8–9), basiventral femoral seta (*bv*) 13 (12–13); genu 4 (3–4), antaxial genual seta (*l’’*) 10 (10–11); tibia 6 (5–6); tarsus 7 (7–8), seta *ft’* 7 (6–7), seta *ft’’* 28 (28–30), seta *u’* 6 (5–6); tarsal empodium (*em*) 7 (6–7), simple, 4-rayed, tarsal solenidion (*ω*) 8 (8–10), slightly knobbed. **Opisthosoma:** opisthosoma dorsally with 35 (35–38) annuli, with round microtubercles at lateral, ventrally with 64 (64–68) annuli, with round microtubercles on rear annular margins. Setae *c2* 23 (23–24) on ventral annulus 12 (12–13), 77 (75–77) apart; setae *d* 45 (45–48) on ventral annulus 25 (24–25), 56 (56–58) apart; setae *e* 23 (20–23) on ventral annulus 39 (39–40), 25 (25–26) apart; setae *f* 25 (24–25) on 5th ventral annulus from rear, 21 (21–22) apart. Setae *h1* 3 (3–4), *h2* 53 (53–65). **Female genitalia** 14 (14–15), 23 (22–23) wide, coverflap with three short lines at base and 12 longitudinal ridges, setae *3a* 17 (17–18), 17 (17–18) apart.

**Male:** Unknown.

**Figure:**
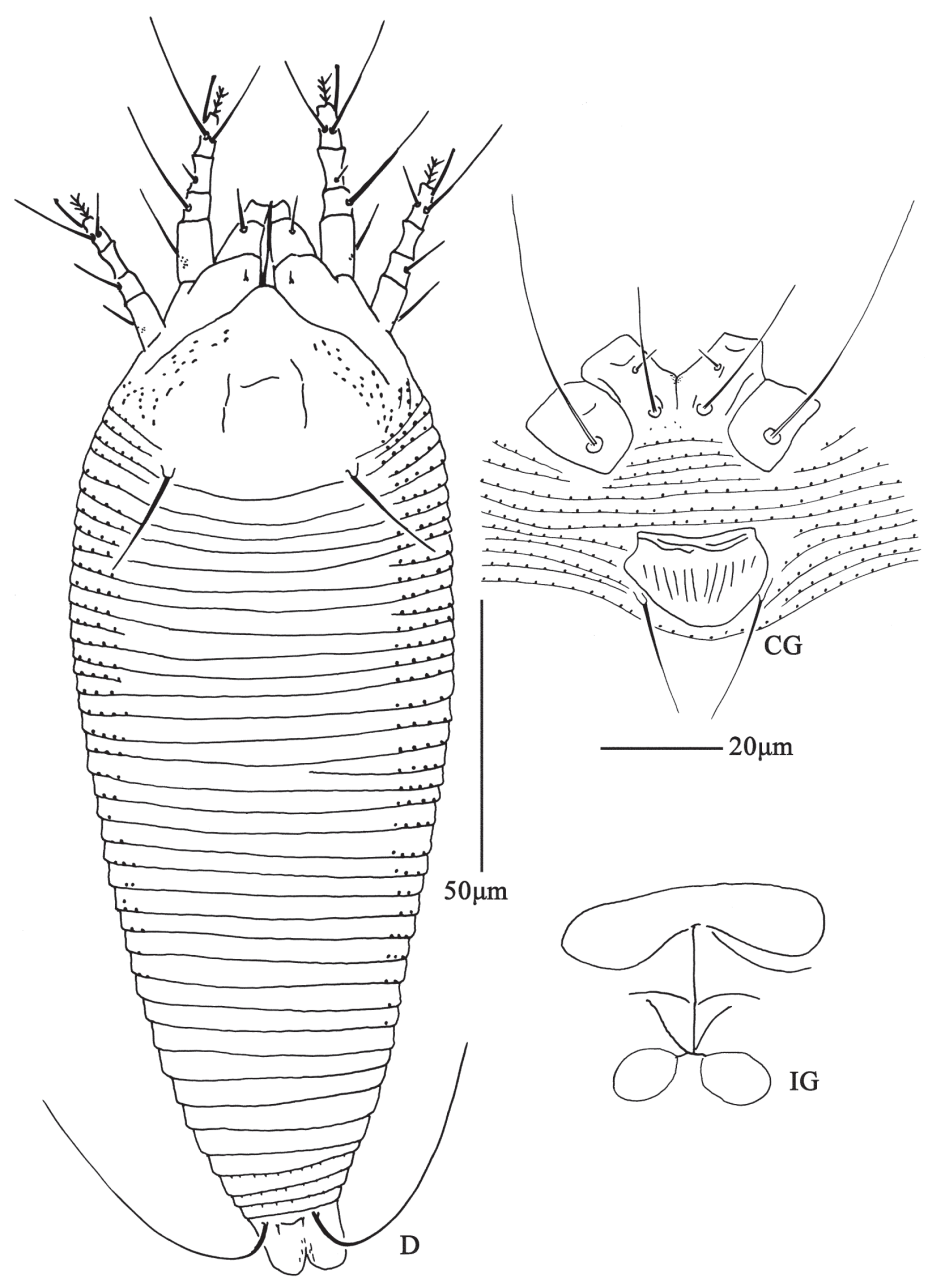
**Figure 12.** *Aculus medicager* sp. n. **D** dorsal view of female **CG** coxae and female genitalia **IG** female internal genitalia.

**Figure:**
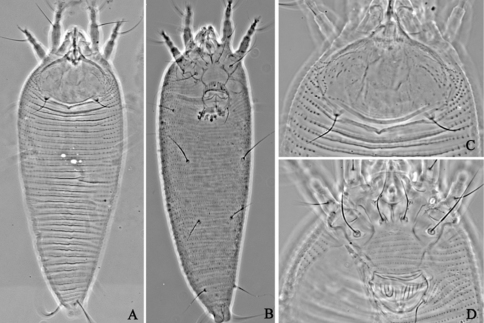
**Figure 14.** *Aculus medicager* sp. n. **A** dorsal view of female **B** ventral view of female **C** prodorsal shield **D** coxae and female genitalia.

##### Type material.

Holotype, female (slide number IRAN204, marked Holotype), from *Medicago sativa* L. (Leguminosae), Ferdowsi University campus, Mashhad, Razavi Khorasan Province, Iran, 36.3000°N, 59.5167°E, elavation 915m, 10-VIII-2010, coll. Samira Sinaie, deposited as slide mounted specimens in the Arthropod/Mite Collection of the Department of Entomology, NJAU, Jiangsu Province, China. Paratypes, 2 females (slide number IRAN204), with the same data as holotype; 11females (slide number 204), from *Medicago sativa* (Leguminosae), Ferdowsi University campus, Mashhad, Razavi Khorasan Province, Iran, 36.3000°N, 59.5167°E, elavation 915m, 10-VIII-2010, coll. Samira Sinaie, deposited in the Department of Plant Protection, FUM, Iran.

##### Relation to host.

Vagrant.

##### Etymology.

The specific designation *medicager* is from the generic name of host plant, *Medicago*.

##### Differential diagnosis.

This species is similar to *Aculus alfalfae* (Roivainen, 1950), (from *Medicago sativa*), but can be differentiated from the latter by prodorsal shield with submedian lines and granules (prodorsal shield smooth in *Aculus alfalfae*), dorsal annuli with granules at lateral (dorsal annuli smooth in *Aculus alfalfae*), empodium 4-rayed (empodium 6-rayed in *Aculus alfalfae*).

##### Remarks.

Alfalfa (*Medicago sativa* L.) is native to Asia Minor. The wild types in the Caucasus and in the mountainous regions of Afghanistan, Iran and adjacent regions. Now, alfalfa is widely cultivated throughout the world as fodder plant for cattle. The new species were described from the local/native plant from Iran.

**Genus *Tetra* Keifer, 1944**

#### Tetra lycopersici

Xue & Hong, 2005 rec. n.

http://species-id.net/wiki/Tetra_lycopersici

Figure 15

Tetra lycopersici Xue & Hong 2005: 46–47, figure 5.

##### Material examined.

3females (slide number IRAN203), from *Solanum nigrum* L. (Solanaceae), Ferdowsi University campus, Mashhad, Razavi Khorasan Province, Iran, 36.3000°N, 59.5167°E, elavation 915m, 30-VII-2010, coll. Samira Sinaie, deposited as slide mounted specimens in the Arthropod/Mite Collection of the Department of Entomology, NJAU, Jiangsu Province, China; 5females and 1 male (slide number 203), from *Solanum nigrum* L. (Solanaceae), Ferdowsi University campus, Mashhad, Razavi Khorasan Province, Iran, 36.3000°N, 59.5167°E, elavation 915m, 30-VII-2010, coll. Samira Sinaie, deposited as slides in the Department of Plant Protection, FUM, Iran.

**Figure:**
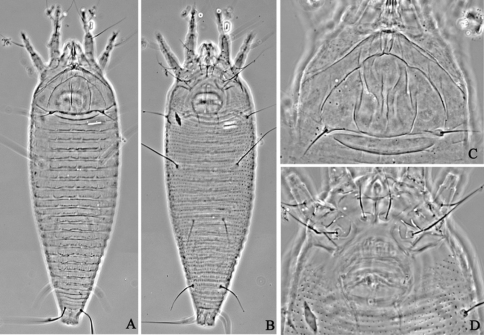
**Figure 15.** *Tetra lycopersici* Xue & Hong, 2005, rec. n. **A** dorsal view of female **B** ventral view of female **C** prodorsal shield **D** coxae and female genitalia.

##### Host.

*Solanum lycopersicum* L. var. *lycopersicon*, *Solanum nigrum* L. (Solanaceae).

##### Relation to host.

Vagrant.

##### Distribution.

China, Iran.

## Supplementary Material

XML Treatment for Aceria acroptiloni

XML Treatment for Aceria anthocoptes

XML Treatment for Aceria lactucae

XML Treatment for Aceria pulicaris

XML Treatment for Aceria tosichella

XML Treatment for Eriophyes rotundae

XML Treatment for Aculops maroccensis

XML Treatment for Aculus medicager

XML Treatment for Tetra lycopersici
